# Supplementary material for: Clinical Effectiveness of Fluorescence Lymph Node Mapping Using ICG for Laparoscopic Right Hemicolectomy: A Prospective Case–Control Study
Source: Cancers (Basel). 2023 Oct 10;15(20):4927. doi: 10.3390/cancers15204927 (PMC10605135; doi:10.3390/cancers15204927)
Supplement: Supplementary file 1 [file cancers-15-04927-s001.zip › cancers-2584142-supplementary.pdf]

Table S1. Comparison of surgical quality according to FLNM (n = 203).

	FLNM group (n = 73)	Control group (n = 130)	p value
Surgical resection specimen length, mean ± SD (range)			
Proximal length (cm)	20.4 ± 13.2 (5.2-77.0)	23.3 ± 14.6 (4.0-104.5)	0.167
Distal length (cm)	18.8 ± 8.6 (5.6-51.1)	19.1 ± 10.0 (1.4-53.3)	0.818
Total length (cm)	43.0 ± 14.8 (18.7-94.7)	46.8 ± 16.8 (18.8-138.1)	0.112
CME quality, n (%)			
Complete CME	73 (100)	128 (98.5)	0.537†
Incomplete CME	0	2 (1.5)	
Harvested lymph node count, mean ± SD (range)			
Pericolic LNs	25.7 ± 12.3 (12-66)	29.2 ± 14.0 (8-90)	0.080
D3 LNs	15.4 ± 8.0 (2-32)	10.3 ± 6.6 (0-33)	< 0.001
ICA LNs	7.5 ± 5.5 (0-21)	5.2 ± 4.7 (0-32)	< 0.001
MCA LNs	7.9 ± 4.8 (0-21)	5.1 ± 4.0 (0-21)	< 0.001
Total LNs	41.1 ± 15.4 (15-98)	39.5 ±16.9 (12-102)	0.499

FLNM; fluorescence lymph node mapping, SD; standard deviation, CME; complete mesocolic excision, LN; lymph node, ICA; ileocolic artery, MCA; middle colic artery, † : Fisher's exact test.

Table S2. Harvested and metastatic lymph nodes count analysis.

(a) Stage I-III patients (*n* = 203)

FLNM group	Harvested LNs				Metastatic LNs			
	n	mean (95% CI)	range	LNs	*n* (%)	mean (95% CI)	range	LNs (%)
pericolic LNs	73	25.7 (22.8, 28.6)	12-66	1877	14 (19.2)	3.5 (1.9, 5.1)	1-9	49 (2.6)
D3 LNs	73	15.4 (13.5, 17.3)	2-32	1124	10 (13.7)	2.4 (1.0, 3.8)	1-7	24 (2.1)
ICA LNs	73	7.5 (6.2, 8.7)	0-21	542	8 (11.0)	2.1 (0.7, 3.6)	1-6	17 (3.1)
MCA LNs	73	8.0 (6.8, 9.1)	0-21	582	4 (5.5)	1.8 (0.2. 3.3)	1-3	7 (1.2)
total LNs	73	41.1 (37.5, 44.7)	15-98	3001	16 (21.9)	4.6 (2.4, 6.7)	1-13	73 (2.4)

Control group	Harvested LNs				Metastatic LNs			
	n	mean (95% CI)	range	LNs	*n* (%)	mean (95% CI)	range	LNs (%)
pericolic LNs	130	29.2 (26.7, 31.6)	8-90	3792	37 (28.5)	3.8 (2.1, 5.5)	1-27	141 (3.7)
D3 LNs	130	10.3 (9.2, 11.5)	0-33	1341	11 (8.5)	3.2 (1.1, 5.2)	1-8	35 (2.6)
ICA LNs	130	5.2 (4.4, 6.0)	0-32	673	6 (4.6)	3.2 (0.3, 0.6)	1-7	19 (2.8)
MCA LNs	130	5.1 (4.4, 5.8)	0-21	668	7 (5.4)	2.3 (0.1, 4.5)	1-7	16 (2.4)
total LNs	130	39.5 (36.5, 42.4)	12-102	5133	37 (28.5)	4.8 (2.7, 6.8)	1-28	176 (3.4)

(b) Stage III patients of FLNM and control group (*n* = 57)

FLNM group	Harvested LNs				Metastatic LNs			
	n	mean (95% CI)	range	LNs	*n* (%)	mean (95% CI)	range	LNs (%)
pericolic LNs	16	31.3 (25.0, 37.7)	12-54	501	14 (87.5)	3.5 (1.9, 5.1)	1-9	49 (9.8)
D3 LNs	16	17.3 (13.8, 20.7)	8-27	276	10 (62.5)*	2.4 (1.0, 3.8)	1-7	24 (8.7)
ICA LNs	16	8.8 (5.8, 11.8)	2-18	141	8 (50.0)*	2.1 (0.7, 3.6)	1-6	17 (12.1)
MCA LNs	16	8.4 (6.4, 10.4)	2-17	135	4 (25.0)	1.8 (0.2, 3.3)	1-3	7 (5.2)
total LNs	16	48.6 (41.9, 55.3)	21-69	777	16 (100)	4.6 (2.4, 6.7)	1-13	73 (9.4)

Control group	Harvested LNs				Metastatic LNs			
	n	mean (95% CI)	range	LNs	*n* (%)	mean (95% CI)	range	LNs (%)
pericolic LNs	41	33.4 (28.2, 38.6)	15-90	1368	37 (90.2)†	3.8 (2.1, 5.5)	1-27	141 (10.3)

D3 LNs	41	9.7 (7.6, 11.8)	1-33	397	11 (26.8)	3.2 (1.1, 5.2)	1-8	35 (8.8)
ICA LNs	41	5.0 (3.1, 6.9)	0-32	205	6 (14.6)	3.2 (0.3, 6.0)	1-7	19 (9.3)
MCA LNs	41	4.7 (3.6, 5.8)	0-13	192	7 (17.1)	2.3 (0.1, 4.5)	1-7	16 (8.3)
total LNs	41	43.1 (37.1, 49.0)	21-102	1765	37 (90.2)†	4.8 (2.7, 6.8)	1-28	176 (10.0)

FLNM; fluorescence lymph node mapping, LN; lymph node, CI; confidence interval, ICA; ileocolic artery, MCA; middle colic artery.

Table S3. Harvested and metastatic lymph nodes in the FLNM group (n = 73).

FLNM group	D3 LNs					
	ICA LNs		MCA LNs		Total	
	n (%)	LNs (%)	*n* (%)	LNs (%)	*n* (%)	LNs (%)
Fluorescent LNs						
Harvested LNs	73	316	73	401	73	717
Metastatic LNs	6 (8.2)	12 (3.8)	3 (4.1)	6 (1.5)	8 (11.0)	18 (2.5)
Non-fluorescent LNs						
Harvested LNs	73	226	73	181	73	407
Metastatic LNs	4 (5.5)	5 (2.2)	1 (1.4)	1 (0.6)	5 (6.8)	6 (1.5)
Total						
Harvested LNs	73	542	73	582	73	1124
Metastatic LNs	8 (11.0)	17 (3.1)	4 (5.5)	7 (1.2)	10 (13.7)	24 (2.1)

FLNM; fluorescence lymph node mapping, LN; lymph node, ICA; ileocolic artery, MCA; middle colic artery
